# Comparison of *Cryptococcus gattii*/*neoformans* Species Complex to Related Genera (*Papiliotrema* and *Naganishia*) Reveal Variances in Virulence Associated Factors and Antifungal Susceptibility

**DOI:** 10.3389/fcimb.2021.642658

**Published:** 2021-07-01

**Authors:** Lana Sarita de Souza Oliveira, Luciana Magalhães Pinto, Mariana Araújo Paulo de Medeiros, Dena L. Toffaletti, Jennifer L. Tenor, Tânia Fraga Barros, Rejane Pereira Neves, Reginaldo Gonçalves de Lima Neto, Eveline Pipolo Milan, Ana Carolina Barbosa Padovan, Walicyranison Plinio da Silva Rocha, John R. Perfect, Guilherme Maranhão Chaves

**Affiliations:** ^1^ Laboratory of Medical and Molecular Mycology, Department of Clinical and Toxicological Analyses, Federal University of Rio Grande do Norte, Natal, Brazil; ^2^ Division of Infectious Disease, Department of Medicine, Duke University School of Medicine, Durham, NC, United States; ^3^ Department of Clinical and Toxicological Analyses, Federal University of Bahia, Salvador, Brazil; ^4^ Department of Mycology, Federal University of Pernambuco, Recife, Brazil; ^5^ Department of Tropical Medicine, Federal University of Pernambuco, Recife, Brazil; ^6^ Department of Infectology, Federal University of Rio Grande do Norte, Natal, Brazil; ^7^ Department of Microbiology and Immunology, Federal University of Alfenas, Alfenas, Brazil; ^8^ Department of Pharmaceutical Sciences, Federal University of Paraiba, João Pessoa, Brazil

**Keywords:** *Cryptococcus* spp., *Papiliotrema laurentii*, *Naganishia* spp., virulence factors, antifungal drug susceptibility

## Abstract

Cryptococcosis is an infectious disease of worldwide distribution, caused by encapsulated yeasts belonging to the phylum Basidiomycota. The genus *Cryptococcus* includes several species distributed around the world. The *C. gattii*/*neoformans* species complex is largely responsible for most cases of cryptococcosis. However, clinical series have been published of infections caused by *Papiliotrema* (*Cryptococcus*) *laurentii* and *Naganishia albida* (*Cryptococcus albidus*), among other related genera. Here, we examined the pathogenic potential and antifungal susceptibility of *C. gattii*/*neoformans* species complex (clades I and II) and related genera (*Papiliotrema* and *Naganishia*) isolated from environmental and clinical samples. *P*. *laurentii* (clade III), *N*. *liquefasciens/N. albidosimilis* (clade IV)**;** and *N. adeliensis*/*N*. *albida* (clade V) strains produced higher levels of phospholipase and hemolysins, whereas the *C. gattii*/*neoformans* species complex strains (clades I and II) had markedly thicker capsules, produced more biofilm biomass and melanin, which are known virulence attributes. Interestingly, 40% of *C*. *neoformans* strains (clade II) had MICs above the ECV established for this species to amphotericin B. Several non-*C. gattii*/*neoformans* species complex (clades III to V) had MICs equal to or above the ECVs established for *C. deuterogattii* and *C. neoformans* for all the three antifungal drugs tested. Finally, all the non-*C. gattii/neoformans* clinical isolates (clades III to V) produced more melanin than the environmental isolates might reflect their particularly enhanced need for melanin during *in vivo* protection. It is very clear that *C. gattii*/*neoformans* species complex (clades I and II) strains, in general, show more similar virulence phenotypes between each other when compared to non-*C. gattii*/*neoformans* species complex (clades III to V) isolates. These observations together with the fact that *P*. *laurentii* and *Naganishia* spp. (clades III to V) strains were collected from the outside of a University Hospital, identify features of these yeasts important for environmental and patient colonization and furthermore, define mechanisms for infections with these uncommon pathogens.

## Introduction

Cryptococcosis is an infectious disease of worldwide distribution, caused by encapsulated yeasts belonging to the phylum Basidiomycota (Kwon-Chung and Bennett, 1992; Maziarz and Perfect, 2016). *Cryptococcus* spp., such as the *Cryptococcus gattii*/*neoformans* species complex, are able to enter mammalian hosts and cause damage to the central nervous system (CNS) (Kwon-Chung et al., 2014; Lakoh et al., 2020) and respiratory tract (Lakoh et al., 2020). In the last few decades, cryptococcal meningoencephalitis has become an infection of global relevance, with up to 1 million new infections annually and significant attributable morbidity and mortality. Even with widespread effective antiretroviral therapy, there is still approximately 200,000 deaths per year especially among patients with human immunodeficiency virus (HIV) infection and AIDS (Park et al., 2009; Park et al., 2011). Besides HIV/AIDS infection, other risk factors for cryptococcosis include corticosteroids and other immunosuppressive therapies, solid organ transplantation, malignant and rheumatological diseases (Maziarz and Perfect, 2016).

The genus *Cryptococcus* includes several species distributed around the world and found in different environments (Chee and Lee, 2005; Loperena-Alvarez et al., 2010), such as bird droppings (mainly pigeons) (Irokanulo et al., 1997; Soltani et al., 2013; Kassi et al., 2018), soil, rivers, decomposing vegetation and tree hollows (Lazera et al., 1998; Kidd et al., 2007; Alves et al., 2016; Santos Bentes et al., 2019). Recently, Hagen et al. (2015) recognized multiple species within the *Cryptococcus gattii*/*neoformans* species complex, including the existence of four *C. neoformans* genotypes: VNI and VNII (var. grubii, serotype A), VNIII (hybrid serotype AD), VNIV (var. neoformans, serotype D); and four genotypes of *C. gattii*: VGI, VGII, VGIII and VGIV (serotypes B and C). Others have also reported the presence of VNBI and VNBII genotypes within the taxon previously considered *C. neoformans* var. *grubii* (Desjardins et al., 2017). More recently, a new *C. gattii* molecular type has been discovered in the African environments of the Central Miombo Woodlands (VGV) (Farrer et al., 2019).

*Cryptococcus gattii*/*neoformans* species complex is primarily related to the vast majority of cases of cryptococcosis (Kwon-Chung et al., 2014; Maziarz and Perfect, 2016), but other species previously known as *C. laurentii* and *C. albidus* can cause disease (Cleveland et al., 2013; Choe et al., 2020). These species are responsible for 80% of the cases of infection caused by non-*Cryptococcus gattii*/*neoformans* species complex yeasts (Johnson et al., 1998; Khawcharoenporn et al., 2007; Smith et al., 2017; Castro-Lainez et al., 2019; Zhang et al., 2019). Based on DNA-sequencing of seven different genes, the class Tremellomycetes (Agaricomicotina lineage, Phylum Basidiomycota) including the genus *Cryptococcus* was recently reclassified. Therefore, the former species *C. laurentii* is now a synonym of *Papiliotrema laurentii* and a few other *Cryptococcus* species of medical interest have been currently included in the genus Naganishia as follows: *Naganishia* (*Cryptococcus) diffluens*, *Naganishia* (*Cryptococcus*) *liquefaciens*, *Naganishia* (*Cryptococcus*) *albidosimilis*, *Naganishia albida* (*Cryptococcus albidus*) and *Naganishia* (*Cryptococcus*) *adeliensis* (Liu et al., 2015).

Several virulence factors have classically been carefully characterized and validated for *C. gattii/neoformans* complex, such as the presence of a polysaccharide capsule that protects yeast cells against phagocytosis, the ability to grow at 37°C, biofilm formation, and the production of additional factors including melanin, phospholipase, metalloprotease, DNase, urease, superoxide dismutase and other antioxidant enzymes (Mancianti et al., 2002; Sanchez et al., 2008; Peng et al., 2018; Zaragoza, 2019). However, only a few studies have investigated the potential role of these and other virulence factors in the non-*Cryptococcus gattii*/*neoformans* species complex (Ikeda et al., 2002; Andrade-Silva et al., 2010; Araujo Gde et al., 2012; Ferreira-Paim et al., 2012; Araujo et al., 2017).

Because of the variations of virulence factors and clinical resistance to azole monotherapy, it is important to know and compare the relevant pathogenic potential and antifungal susceptibility profiling of *Cryptococcus* spp. collected from clinical and environmental sources. Therefore, the primary objectives of this study were to evaluate the pathogenic features and antifungal susceptibility of *Cryptococcus*-related genera isolated from pigeon droppings (environmental), compared to the *Cryptococcus gattii*/*neoformans* species complex strains obtained from clinical samples of patients belonging to 3 different Brazilian states (Bahia, Pernambuco and Rio Grande do Norte) in the Northeast region. This is the first study to demonstrate that non-*Cryptococcus gattii*/*neoformans* species complex may express a full range of known virulence factors *in vitro* and furthermore, some strains may exhibit increased minimal inhibitory concentrations (MICs) to antifungal drugs without previous known exposure to these compounds.

## Materials and Methods

### Collection and Processing of Guano Samples From Pigeons

The study included samples of pigeon droppings collected outside the building of a tertiary care university hospital, located in Natal City, Rio Grande do Norte State, Brazil, from April 2012 to March 2014 and November 2017 to February 2018.

Approximately, 2g of dried and/or fresh pigeon guano was collected from several sites and diluted in saline solution (0.9% NaCl) in a ratio of 1:10, vortexed for 10 minutes and allowed to stand for 1h. Subsequently, 100 μL of the supernatant was inoculated on the surface of Sabouraud Dextrose Agar (SDA; Oxoid, Basingstoke, Hampshire, UK) and Rose Bengal (0.05 g/L) plates with the aid of a Drigalski loop. Each plate was incubated at 30°C for 48h. Yeast colonies with creamy to mucoid phenotypes were selected for subsequent identification.

### Phenotypic Identification of *Cryptoccocus* spp. Related Genera Isolates

After colony growth, yeast cells were streaked onto the surface of CHROMagar *Candida* ^®^ (CHROMagar Microbiology, Paris, France) to check for purity and screening for different color colonies (Baumgartner et al., 1996). The initial identification (screening for the *Cryptococcus* genus) was based on the characteristics of the yeast cells observed microscopically after cultivation on corn meal agar with Tween 80, glucose fermentation (non-fermentative), urease test, Niger seed agar (NSA) for melanin production and capsule visualization with an India ink stain (Yarrow, 1998). After identification, all cryptococcal yeast isolates were cultured in YPD broth (Yeast Peptone Dextrose, yeast extract 10 g/L, dextrose 20g/L, peptone 20g/L) overnight at 30°C, and then transferred to cryotubes containing 20% glycerol and stored at -80°C. The isolates now belong to the fungal culture collection of the Medical and Molecular Mycology Laboratory, Department of Clinical and Toxicological Analyses, Federal University of Rio Grande do Norte.

### Molecular Identification of *Cryptoccocus* spp. and Related Genera Isolates

#### DNA Extraction

The isolates were grown on YPD (BD Difco, NJ, USA) plates for 72 h and then, a single colony was inoculated into 3.5 mL of 2xYPD broth in a 14 ml culture tube and incubated at 30°C with shaking (225 rpm) for 48 h. DNA was extracted using the MasterPure Yeast DNA Purification Kit (Epicentre Biotechnologies, Madison, WI, USA). DNA concentrations were measured using the Qubit dsDNA HS assay according to instructions (Life Technologies).

#### PCR Assay and ITS Region Sequencing

The PCR Hot Start Taq 2X Master Mix (New England Biolabs) was used to amplify all fragments generated in this work. Samples were amplified in a Bio-rad thermocycler (Thermo Cycler T100) using the following cycling parameters for both regions: one initial cycle of 95°C for 30 sec; followed by 30 cycles of 30 sec at 95°C, 1 min at 47°C and 1 min at 68°C and a final cycle of 5 min at 68°C. PCR products were treated with ExoSAP-IT (Thermo Fisher Scientific) according to the manufacturer’s protocol. Purified PCR products were sequenced by the Sanger sequencing method on ABI 3730 xl DNA Sequencers by Eton Bioscience, Inc. For sequencing the ITS region, the internal primers ITS1 and ITS4 were used (White et al., 1990). Nucleotide sequences were submitted for BLAST analysis at the NCBI site (http://www.ncbi.nlm.nih.gov) for species identification. Only sequences deposited in GenBank showing high similarities with our query sequences and an E value of lower than 10^−5^ were used in this study.

#### Genbank Accession Numbers

GenBank accession numbers may be seen in **Table 1**.

**Table 1:** Phenotypic properties and virulence attributes determined *in vitro* of clinical and environmental isolates of *Cryptococcus* spp. and related genera obtained from patients and PD in Northeast Brazil.

Strain name	GenBank accession number	State/Source	Aspect on SDA*/Chromagar *Candida* ^®^ color	Growth at 37°C	NSA**	Phospholipase zone (Pz)	Hemolysis Index (HI)	Capsule thickness (µm)	Biofilm Formation (O.D._570nm_)
*C. deuterogattii* (clade I)									
LMMM^1^696	MT409611	RN^2^/CSF^5^	Mucoid/Purple	+	3	0.47 ± 0.03	Negative	9.87 ± 3.13	1.12 ± 0.03
LMMM700	MT409595	RN/CSF	Mucoid/Lilac	+	2	0.72 ± 0.10	Negative	8.63 ± 1.29	1.14 ± 0.06
LMMM701	MT409604	RN/CSF	Mucoid/Purple	+	1	0.71 ± 0.06	0.55 ± 0.06	8.66 ± 1.34	1.05 ± 0.05
LMMM702	MT409605	RN/CSF	Mucoid/Lilac	+	3	0.67 ± 0.09	Negative	10.16 ± 1.87	0.75 ± 0.08
LMMM764	MT409600	RN/CSF	Creamy/Purple	+	1	0.79 ± 0.03	0.47 ± 0.04	21.81 ± 5.65	1.13 ± 0.08
LMMM1271	MT409606	RN/CSF	Mucoid/Pink	+	1	0.76 ± 0.07	Negative	13.74 ± 3.48	1.10 ± 0.04
LMMM681	MT409596	RN/CSF	Mucoid/Purple	+	4	0.66 ± 0.03	Negative	10.96 ± 2.25	1.12 ± 0.08
LMMM1241	MT409601	RN/BL	Mucoid/Pink	+	3	0.70 ± 0.00	0.71 ± 0.06	35.87 ± 7.33	0.63 ± 0.03
LMMM1267	MT409609	RN/CSF	Mucoid/Lilac	+	4	0.74 ± 0.04	0.62 ± 0.09	11.07 ± 1.82	1.02 ± 0.06
LMMM1397	MT409592	RN/CSF	Creamy/Lilac	+	4	0.59 ± 0.09	0.46 ± 0.06	4.1 ± 1.27	1.13 ± 0.06
LMMM1237	MT409597	RN/CSF	Creamy/Purple	+	4	0.33 ± 0.03	0.54 ± 0.02	13.36 ± 2.38	1.12 ± 0.03
LMMM770	MT409598	BA^3^/CSF	Mucoid/Pink	+	1	0.52 ± 0.08	0.52 ± 0.03	23.2 ± 3.28	1.13 ± 0.03
LMMM771	MT409602	BA/CSF	Mucoid/Lilac	+	4	0.54 ± 0.09	0.55 ± 0.02	27.17 ± 3.4	1.09 ± 0.03
LMMM774	MT409607	BA/CSF	Mucoid/Purple; Dark blue	+	2	0.75 ± 0.05	0.69 ± 0.03	25.02 ± 4.77	1.11 ± 0.05
LMMM779	MT409593	BA/CSF	Creamy/Purple	+	3	0.66 ± 0.01	Negative	24.65 ± 4.22	1.10 ± 0.04
LMMM782	MT409603	BA/CSF	Mucoid/Pink	+	1	0.75 ± 0.06	0.52 ± 0.02	30.61 ± 4.55	1.12 ± 0.03
LMMM786	MT409608	BA/CSF	Mucoid/Lilac	+	3	0.65 ± 0.05	0.51 ± 0.04	30.41 ± 5.59	0.85 ± 0.05
LMMM787	MT409594	BA/CSF	Creamy/Lilac	+	1	0.47 ± 0.06	0.60 ± 0.08	36.07 ± 9.33	1.12 ± 0.09
LMMM788	MT409610	BA/CSF	Mucoid/Purple	+	2	0.43 ± 0.06	Negative	27.17 ± 5.02	1.09 ± 0.05
LMMM789	MT409599	BA/CSF	Mucoid/Purple; Dark Blue	+	2	0.42 ± 0.02	0.48 ± 0.02	22.79 ± 5.19	1.02 ± 0.07
*C. neoformans* (clade II)									
LMMM763	MT409227	RN/CSF	Creamy/Pink	+	2	0.63 ± 0.02	0.52 ± 0.02	12.04 ± 1.59	0.92 ± 0.04
LMMM767	MT412413	RN/CSF	Creamy/Pink	+	4	0.71 ± 0.07	0.51 ± 0.02	12.36 ± 2.9	0.93 ± 0.07
LMMM768	MT412431	RN/CSF	Mucoid/Pink	+	4	0.57 ± 0.05	0.68 ± 0.04	18.04 ± 3.28	0.90 ± 0.05
LMMM769	MT412436	RN/CSF	Creamy/Pink	+	4	0.64 ± 0.06	0.63 ± 0.06	10.83 ± 3.56	0.83 ± 0.06
LMMM695	MT412421	RN/CSF	Creamy/Pink	+	2	0.72 ± 0.06	0.51 ± 0.07	9.56 ± 2.47	0.82 ± 0.02
LMMM619	MT412411	RN/CSF	Creamy/Pink	+	3	0.73 ± 0.04	0.53 ± 0.06	10 ± 1.92	1.07 ± 0.04
LMMM679	MT412422	RN/CSF	Creamy/Pink	+	2	0.71 ± 0.02	0.47 ± 0.10	9.77 ± 1.64	0.95 ± 0.07
LMMM680	MT412434	RN/CSF	Creamy/Pink	+	3	0.63 ± 0.03	0.56 ± 0.03	5.07 ± 1.19	0.90 ± 0.06
LMMM682	MT409235	RN/CSF	Creamy/Lilac	+	3	0.60 ± 0.06	0.49 ± 0.05	5.41 ± 0.77	0.84 ± 0.07
LMMM683	MT412412	RN/CSF	Mucoid/Pink	+	4	0.56 ± 0.04	0.56 ± 0.09	9.84 ± 1.72	0.95 ± 0.05
LMMM684	MT412430	RN/CSF	Creamy/Pink	+	4	0.61 ± 0.03	0.54 ± 0.04	10.99 ± 2.01	0.73 ± 0.06
LMMM685	MT412435	RN/CSF	Creamy/Pink	+	2	0.54 ± 0.02	0.41 ± 0.02	10.27 ± 1.49	0.80 ± 0.04
LMMM222	MT409226	RN/Urine	Creamy/Purple	+	2	0.57 ± 0.01	0.60 ± 0.06	8.53 ± 1.92	0.63 ± 0.05
LMMM620	MT409229	PE^4^/CSF	Mucoid/Pink	+	2	0.82 ± 0.03	0.63 ± 0.02	20.95 ± 5.28	0.93 ± 0.06
LMMM621	MT412442	PE/CSF	Mucoid/Pink	+	0	0.72 ± 0.03	0.74 ± 0.07	6.36 ± 1.03	0.90 ± 0.05
LMMM622	MT409231	PE/CSF	Mucoid/Pink	+	3	0.71 ± 0.03	0.64 ± 0.03	14.24 ± 1.94	0.93 ± 0.08
LMMM623	MT412409	PE/CSF	Mucoid/Pink	+	3	0.71 ± 0.02	0.65 ± 0.04	15.84 ± 2.48	0.90 ± 0.04
LMMM624	MT412420	PE/CSF	Mucoid/Pink	+	0	0.72 ± 0.04	0.69 ± 0.03	16.4 ± 3.28	1.01 ± 0.004
LMMM625	MT409236	PE/CSF	Mucoid/Lilac	+	4	0.59 ± 0.02	0.66 ± 0.03	16.63 ± 2.52	0.86 ± 0.06
LMMM626	MT412443	PE/CSF	Mucoid/Pink	+	4	0.73 ± 0.04	0.66 ± 0.03	18.17 ± 3.24	0.92 ± 0.04
LMMM627	MT412444	PE/CSF	Mucoid/Pink	+	4	0.72 ± 0.07	0.69 ± 0.05	18.7 ± 2.6	0.92 ± 0.03
LMMM628	MT412419	PE/CSF	Mucoid/Pink	+	4	0.59 ± 0.02	0.67 ± 0.02	17.87 ± 4.19	0.99 ± 0.09
LMMM629	MT412417	PE/CSF	Mucoid/Pink	+	1	0.69 ± 0.03	0.65 ± 0.04	15.88 ± 2.62	0.93 ± 0.04
LMMM630	MT409230	PE/CSF	Mucoid/Pink	+	4	0.67 ± 0.01	0.71 ± 0.03	17.21 ± 3.48	0.98 ± 0.06
LMMM631	MT409222	PE/CSF	Mucoid/Pink	+	4	0.68 ± 0.03	0.65 ± 0.02	18.59 ± 4.2	0.99 ± 0.07
LMMM632	MT412439	PE/CSF	Mucoid/Pink	+	4	0.68 ± 0.02	0.59 ± 0.03	19.57 ± 2.69	0.88 ± 0.09
LMMM633	MT409234	PE/CSF	Mucoid/Lilac	+	3	0.73 ± 0.03	0.78 ± 0.07	13.69 ± 2.24	0.98 ± 0.07
LMMM634	MT412437	PE/CSF	Mucoid/Pink	+	3	0.73 ± 0.03	0.65 ± 0.02	16.18 ± 2.44	0.75 ± 0.04
LMMM635	MT412438	PE/CSF	Mucoid/Pink	+	4	0.78 ± 0.03	0.63 ± 0.03	20.07 ± 2.44	0.89 ± 0.08
LMMM636	MT412441	PE/CSF	Mucoid/Pink	+	3	0.72 ± 0.04	0.60 ± 0.05	18.23 ± 3.36	0.95 ± 0.04
LMMM637	MT412415	PE/CSF	Mucoid/Pink	+	4	0.64 ± 0.05	0.67 ± 0.04	18.42 ± 3.69	1.02 ± 0.02
LMMM638	MT409233	PE/CSF	Mucoid/Pink	+	1	0.83 ± 0.05	0.51 ± 0.01	11.6 ± 1.78	0.94 ± 0.02
LMMM639	MT412416	PE/CSF	Mucoid/Pink	+	4	0.73 ± 0.03	0.61 ± 0.05	18.46 ± 4.45	1.00 ± 0.02
LMMM640	MT409223	PE/CSF	Mucoid/Pink	+	3	0.73 ± 0.05	0.71 ± 0.03	18.02 ± 3.62	0.73 ± 0.01
LMMM641	MT409225	PE/CSF	Mucoid/Pink	+	4	0.71 ± 0.03	0.61 ± 0.03	16.46 ± 2.42	1.03 ± 0.08
LMMM642	MT412440	PE/CSF	Mucoid/Pink	+	3	0.75 ± 0.05	0.65 ± 0.03	19.17 ± 3.43	0.84 ± 0.01
LMMM643	MT412408	PE/CSF	Mucoid/Pink	+	3	0.54 ± 0.05	0.62 ± 0.04	19.08 ± 2.92	1.07 ± 0.05
LMMM644	MT412418	PE/CSF	Mucoid/Pink	+	4	0.83 ± 0.04	0.64 ± 0	19.13 ± 4.45	0.45 ± 0.09
LMMM645	MT412433	PE/CSF	Mucoid/Pink	+	0	0.55 ± 0.05	0.69 ± 0.03	18.03 ± 3.8	0.85 ± 0.07
LMMM646	MT412414	PE/CSF	Mucoid/Pink	+	4	0.63 ± 0.04	0.59 ± 0.02	16.28 ± 3.44	0.93 ± 0.04
LMMM647	MT409224	PE/CSF	Mucoid/Pink	+	4	0.55 ± 0.05	0.64 ± 0.07	16.81 ± 3.53	1.00 ± 0.08
LMMM648	MT409232	PE/CSF	Mucoid/Pink	+	4	0.80 ± 0.01	0.68 ± 0.07	18.41 ± 4.02	1.01 ± 0.02
LMMM649	MT412410	PE/CSF	Mucoid/Pink	+	1	0.82 ± 0.03	0.65 ± 0.04	10.02 ± 2.01	0.82 ± 0.05
LMMM777	MT412432	BA/CSF	Mucoid/Pink	+	3	0.60 ± 0.04	0.52 ± 0.08	19.53 ± 2.57	0.95 ± 0.09
LMMM780	MT409228	BA/CSF	Creamy/Purple	+	3	0.59 ± 0.02	0.53 ± 0.08	12.53 ± 2.27	1.01 ± 0.02
*P. laurentii* (clade III)									
LMMM1422	MT462593	RN/PD	Creamy and Pink/Purple	–	0	0.48 ± 0.05	0.44 ± 0.02	4.87 ± 0.71	0.54 ± 0.06
LMMM1423	MT462585	RN/PD	Creamy/Purple	–	1	0.59 ± 0.07	Negative	4.93 ± 0.83	0.63 ± 0.03
LMMM1424	MT462588	RN/PD	Mucoid/Purple	–	0	0.52 ± 0.07	0.66 ± 0.06	5.34 ± 0.76	0.54 ± 0.05
LMMM1426	MT462591	RN/PD	Mucoid/Purple	–	0	0.52 ± 0.08	0.57 ± 0.04	5.72 ± 1.05	0.43 ± 0.04
LMMM1431	MT462589	RN/PD	Creamy and Pink/Purple	–	1	0.59 ± 0.03	0.46 ± 0.04	6.72 ± 1.22	0.48 ± 0.02
LMMM1432	MT462590	RN/PD	Mucoid and Pink/Purple	–	1	0.78 ± 0.03	0.53 ± 0.08	6.35 ± 1.11	0.43 ± 0.05
LMMM1433	MT462586	RN/PD	Mucoid/Purple	–	1	0.61 ± 0.04	0.54 ± 0.04	5.88 ± 1.14	0.44 ± 0.05
LMMM1435	MT462592	RN/PD	Mucoid/Purple	–	1	0.33 ± 0.01	0.50 ± 0.04	9.58 ± 2.59	0.46 ± 0.02
LMMM1436	MT462584	RN/PD	Mucoid and Pink/Purple	–	1	0.68 ± 0.16	0.73 ± 0.06	4.54 ± 0.87	0.55 ± 0.04
LMMM1398	MT462587	RN/CSF	Creamy/Purple	–	3	0.45 ± 0.05	0.54 ± 0.02	15.82 ± 2.72	0.51 ± 0.02
*N. liquefasciens/N. Albidosimilis* (clade IV)									
LMMM388	MT462598	RN/PD	Creamy and Pink/Purple	–	1	0.53 ± 0.06	Negative	4.59 ± 1.1	0.39 ± 0.05
LMMM211	MT462599	RN/PD	Mucoid/Purple	–	1	0.56 ± 0.05	0.43 ± 0.03	9.09 ± 2.14	0.36 ± 0.06
LMMM1434	MT462600	RN/PD	Creamy/Purple	–	1	0.50 ± 0.06	0.57 ± 0.04	7.94 ± 1.85	0.37 ± 0.07
LMMM460	MT462601	RN/PD	Mucoid and Pink/Purple	–	0	0.27 ± 0.02	0.43 ± 0.03	6.7 ± 1.54	0.35 ± 0.04
LMMM400	MT462602	RN/PD	Mucoid and Pink/Purple	–	1	0.30 ± 0.05	0.51 ± 0.05	11.7 ± 1.54	0.42 ± 0.08
LMMM374	MT462605	RN/PD	Creamy and Pink/Purple	–	1	0.35 ± 0.03	0.55 ± 0.04	5.25 ± 1.15	0.34 ± 0.02
LMMM383	MT462604	RN/PD	Mucoid/Purple	–	1	0.36 ± 0.05	0.37 ± 0.06	6.84 ± 1.52	0.52 ± 0.05
LMMM385	MT462610	RN/PD	Creamy/Purple	–	1	0.32 ± 0.02	0.48 ± 0.02	4.73 ± 0.99	0.35 ± 0.04
LMMM386	MT462613	RN/PD	Mucoid/Purple	–	1	0.32 ± 0.03	0.38 ± 0.07	4.98 ± 1.2	0.47 ± 0.03
LMMM387	MT462616	RN/PD	Mucoid and Pink/Purple	–	1	0.41 ± 0.02	0.53 ± 0.03	6.43 ± 1.76	0.52 ± 0.06
LMMM389	MT462606	RN/PD	Mucoid and Pink/Purple	–	1	0.63 ± 0.04	0.44 ± 0.04	4.65 ± 1.07	0.54 ± 0.04
LMMM423	MT462611	RN/PD	Mucoid/Purple	–	1	0.53 ± 0.05	0.42 ± 0.03	10.01 ± 3.89	0.47 ± 0.03
LMMM170	MT462612	RN/PD	MucoidandCreamy/Purple	–	1	0.31 ± 0.02	0.42 ± 0.03	13.19 ± 3.67	0.35 ± 0.02
LMMM171	MT462615	RN/PD	Mucoid and Pink/Purple	–	1	0.28 ± 0.01	0.41 ± 0.05	10.48 ± 1.45	0.34 ± 0.02
LMMM175	MT462609	RN/PD	Creamy/Purple	–	1	0.34 ± 0.04	0.49 ± 0.07	10.2 ± 2.08	0.45 ± 0.04
LMMM176	MT462603	RN/PD	Creamy and Pink/Purple	–	1	0.39 ± 0.05	0.46 ± 0.04	9.53 ± 1.6	0.38 ± 0.03
LMMM463	MT462608	RN/PD	Mucoid/Purple	–	1	0.29 ± 0.01	0.46 ± 0.04	12.97 ± 2.36	0.39 ± 0.04
LMMM219	MT462614	RN/PD	Creamy and Pink/Purple	–	0	0.59 ± 0.06	0.42 ± 0.06	9.03 ± 1.79	0.29 ± 0.03
LMMM1425	MT462617	RN/PD	Mucoid and Pink/Purple	–	1	0.38 ± 0.02	0.48 ± 0.03	7.43 ± 3.79	0.38 ± 0.05
LMMM1460	MT462607	RN/CSF	Creamy/Purple	–	3	0.60 ± 0.09	0.46 ± 0.10	3.13 ± 0.76	0.49 ± 0.03
*N. albida/N. adeliensis* (clade V)									
LMMM167	MT462622	RN/PD	Creamy and Pink/Pink	–	1	0.46 ± 0.01	0.38 ± 0.05	9.13 ± 1.79	0.39 ± 0.05
LMMM1428	MT462621	RN/PD	Creamy/Lilac	–	1	0.49 ± 0.03	0.45 ± 0.02	8.53 ± 1.88	0.52 ± 0.04
LMMM1427	MT462619	RN/PD	Mucoid/Purple	–	1	0.42 ± 0.03	0.47 ± 0.06	10.79 ± 4.46	0.52 ± 0.06
LMMM1430	MT462618	RN/PD	Opaque/Lilac	–	1	0.46 ± 0.05	0.48 ± 0.02	9.26 ± 2.05	0.45 ± 0.07
LMMM425	MT462620	RN/PD	Creamy and Pink/Purple	–	1	0.33 ± 0.03	Negative	8.97 ± 1.61	0.38 ± 0.06
LMMM406	MT462623	RN/PD	Creamy and Pink/Lilac	–	1	0.43 ± 0.06	0.83 ± 0.05	29.56 ± 3.82	0.21 ± 0.03
LMMM411	MT462624	RN/PD	Creamy/Pink	–	0	0.29 ± 0.01	0.42 ± 0.08	16.44 ± 7.69	0.31 ± 0.01
LMMM390	MT462625	RN/PD	Creamy and Pink/Lilac	–	1	0.33 ± 0.04	0.45 ± 0.05	3.98 ± 0.83	0.43 ± 0.02
LMMM459	MT462628	RN/PD	Creamy and Pink/Purple	–	1	0.47 ± 0.08	0.52 ± 0.04	12.97 ± 1.94	0.45 ± 0.05
LMMM1429	MT462627	RN/PD	Creamy/Lilac	–	1	0.45 ± 0.01	0.46 ± 0.05	8.85 ± 2.39	0.39 ± 0.08
LMMM221	MT462626	RN/CSF	Opaque/Lilac	–	3	0.69 ± 0.08	0.56 ± 0.06	6.78 ± 1.27	0.50 ± 0.05

*Sabouraud Dextrose Agar, **Niger Seed Agar, 1-Laboratory of Medical and Molecular Mycology, 2-Rio Grande do Norte State, 3-Bahia State, 4-Pernambuco state, 5-Cerebrospinal fluid. Gray dark dashed borders stand for strong production, whereas light grey means moderate production. Non-colored numbers mean weak or negative expression of virulence factors. BL, Bronchial lavage; PD, Pigeon droppings.

#### Phylogenetic Analysis

The ITS rDNA sequences generated from all the isolates were aligned with type strain sequences from *Cryptococcus* spp. and the other genera present as matches in the BLASTn searches (http://blast.ncbi.nlm.nih.gov/Blast.cgi), according to taxonomic reclassification of members within the Tremellomycetes (Liu et al., 2015). All sequences were aligned using the Muscle algorithm implemented by MEGA X (Kumar et al., 2018). The phylogeny was inferred using the Neighbor-Joining method (Saitou and Nei, 1987), including gap positions, and the evolutionary distances were corrected by the Kimura 2-parameter method (Kimura, 1980). Bootstrap analysis (bt) (Felsenstein, 1985) was conducted by evaluating 1,000 pseudoreplicates of the alignment. All analyses were computed using MEGA X (Kumar et al., 2018).

### Isolates Used in the Present Study

We have included 106 isolates of *Cryptococcus* spp. and related genera from our culture collection to perform the evaluation of these isolates virulence factor expressions and antifungal susceptibility testing *in vitro*. Thirty-eight of them were environmental: *Papiliotrema laurentii* (clade III; n=9), *N*. *liquefasciens/N. albidosimilis* (clade IV; n=19), *N. adeliensis*/*N*. *albida* (clade V; n=10). Sixty-eighth clinical isolates primarily from the *Cryptococcus gattii*/*neoformans* species complex were assessed including *C. neoformans* (clade II; n=45), *C. gattii* (clade I; n=20), *P. laurenttii* (clade III; n=1), *N*. *liquefasciens/N. albidosimilis* (clade IV; n=1) and *N. adeliensis*/*N*. *albida* (clade V; n=1). The clinical isolates were obtained from patients admitted to tertiary hospitals from three different Brazilian states (Rio Grande do Norte, Pernambuco and Bahia); all of them located in the Brazilian Northeast region (**Table 1**).

### Inoculum Preparation of *Cryptococcus* spp. and Related Genera Isolates for *In Vitro* Assessment of Virulence Factors

For the phenotypic characterizations of the different isolates, the yeasts were initially grown in NGY broth medium (Difco Neopeptone 1 g/L, Dextrose 4 g/L; Difco yeast extract 1 g/L) prior to the phospholipase and hemolysin enzymatic activity tests. Yeast cells were inoculated by “wet looping” in this medium with a ring loop loaded with a yeast suspension film, rapidly immersed in the medium and removed and incubated for 18-24h in a shaker incubator at 30°C at 200 rpm. An inoculum of approximately 2x10^8^ cells/mL is produced (Chaves et al., 2007). Cultures were spectrophotometrically measured at a wavelength of 600nm ranging from 0.8 and 1.2 (Biochrom Libra S32). Subsequently, yeast cells were diluted to obtain the same inoculum for each condition evaluated *in vitro*.

### *Cryptococcus* spp. and Related Genera for Phospholipase Production

For detection of the phospholipase activity, the method of Price et al. (1982) (Price et al., 1982) adapted for *Cryptococcus* spp. was used (Pini et al., 2017). Overnight NGY cultures were diluted and standardized to a concentration of 2x10^8^ yeast cells/mL and the suspension of yeast cells was inoculated in triplicate onto the surface of phospholipase agar (10 g peptone, 40 g dextrose, 16 g agar, 80 mL Egg Yolk Emulsion [Fluka] was added to 1000 mL of distilled water). The plates were incubated at 30°C for 10 days. After the incubation period, the diameters of the colonies and the halo formed around them were measured. The Pz (phospholipase zone) was determined by dividing the colony diameter by the precipitation zone plus colony diameter. The strains were classified as follows, according to tertiles distribution: Pz = 1 as negative phospholipase activity; 0.65 ≤ Pz ≤ 0.83 as weak; 0.46 ≤ Pz ≤ 0.64 as moderate; 0.27 ≤ Pz ≤ 0.45 as strong phospholipase producers.

### Measurement of Hemolysin Production of *Cryptococcus* spp. and Related Genera

In order to evaluate hemolysin production, we followed the methodology proposed by Luo (Luo et al., 2001) with some adaptations. Yeast cells were initially cultured on SDA at 30°C for 24 h. Strains were next grown overnight in NGY broth. Ten microliters of the culture were streaked out in triplicate onto the surface of SDA containing 7% fresh sheep blood (Ebe-Farma) and 3% glucose, in Petri dishes of 155 mm of diameter. The plates were incubated for 48 h at 37°C in an atmosphere with 5% CO_2_. After the incubation period, the presence of a clear halo around the inoculum indicated positive hemolysis. The diameter of colonies and zones of hemolysis were measured in order to obtain the hemolysis index (HI) for each strain. HI was determined by dividing the colony diameter by the precipitation zone plus colony diameter, which allowed classification of isolates into strong, moderate and weak producers. The strains were classified as follows, according to tertiles distribution: HI= 1 as negative hemolytic index; 0.69 ≤ Pz ≤ 0.84 as weak; 0.53 ≤ HI ≤ 0.68 as moderate; 0.37 ≤ HI ≤ 0.52 as strong hemolysin producers.

### Capsule Size Measurement in *Cryptococcus* spp. and Related Genera

To perform the capsule experiments, we used the protocol of Zaragoza et al., (Zaragoza et al., 2003) with adaptations. All the strains were grown in YPD broth for 24 h at 37°C with 200 rpm agitation. After washing with phosphate buffer saline (PBS), yeast cells were adjusted to 1×10^5^ cells/mL using a hemocytometer to standardize the inoculum and inoculated in a solution containing 10% fetal bovine serum (FBS; Sigma-Aldrich; Missouri, Saint Louis, USA) diluted in PBS (5mL of final solution in conical tubes). The tubes were incubated for 24 h at 37°C in an atmosphere of 5% CO_2_. Subsequently, a drop of India ink stain was added to a small volume of yeast cells in suspension and placed on a microscope slide. The samples were imaged with a Nikon Eclipse E100 microscope. Pictures were taken using a Tsview 7 software. Total diameter (including capsule) (dt) and yeast cell diameter (dy) were measured for each strain. Capsule thickness (tc) was determined with the following equation: tc=1/2 (dt-dy) in micrometers (Fernandes et al., 2018).

### Melanin Production in *Cryptococcus* spp. and Related Genera

To evaluate melanin production, yeast cells were cultured on SDA for 72 h at 30°C. Then, a single colony was streaked out thrice (parallel sides) on the surface of NSA medium at 30°C for 10 days for visualization of melanin production. Scores from 0 to 4 were attributed as follows: 0 (zero), no pigment; 1, light brown; 2,brown; 3, dark brown; and 4, for almost black colonies (Pedroso et al., 2009).

### Biofilm Formation of *Cryptococcus* spp. and Related Genera

Biofilm formation assays were performed according to Palanco et al. (2017) with some modifications. The strains were previously cultivated in Sabouraud broth at 30°C, for 24 h with shaking at 200rpm. Then, yeast cells were washed twice with PBS and 100 μL aliquots of a standardized cell suspension (1 x 10^8^ cells/mL)were transferred to flat bottom 96 well microtiter plates and incubated for 4 h at 37°C for the adhesion phase. As controls, eight wells of each microtiter plate were handled similarly, except that no yeast cell suspensions were added. Following the adhesion phase, cell suspensions were aspirated and each well was washed twice with 150 μL of PBS to remove loosely adherent cells. A total of 200 μL of BHI (Brain Heart Infusion) medium (BD Difco) was added to each of the washed wells and incubated at 37°C in a shaker incubator at 100 rpm. Biofilms were allowed to develop for 48 h and quantified by a crystal violet assay. Briefly, the biofilm-coated wells of the microtiter plates were washed twice with 150 μL of PBS and then air dried. Subsequently, each of the washed wells was stained with 100 μL of 0.4% aqueous crystal violet solution for 5 min. Subsequently, each well was washed once with 150 μL sterile distilled water and immediately distained with the addition of 100 μL of 95% ethanol for approximately 1 min until complete crystal violet solubilization. Subsequently, 100 μL of the destaining solution was transferred to a clean well and the amount of the crystal violet stain in the referred solution was measured with a microtiter plate reader (Spectra MAX 340 Tunable Microplate Reader; Molecular Devices Ltda.) at 570 nm. The absorbance values for the controls were subtracted from the values from the test wells to minimize background interference. The isolates were classified as follows, according to tertiles distribution: 0.21 ≤ OD570nm ≤ 0.52 as weak; 0.53≤ OD570nm ≤ 0.84 as moderate; 0.85 ≤ OD570nm ≤ 1.16 as strong biofilm producers.

### Antifungal Susceptibility Profile of *Cryptococcus* spp. and Related Genera

Solutions of fluconazole (FLU), itraconazole (ITC) and amphotericin B (AMB) were prepared in accordance with guidelines M27-A3 (CLSI, 2008) by being diluted in RPMI 1640 (Roswell Park Memorial Institute; Angus buffers and Biochemical, Niagara Falls, NY, USA) buffered 3-(N-morpholino) propanesulfonic acid (MOPS) to pH 7.0. Antifungal drugs tested were diluted serially in 10 different concentrations, namely: FLU (Pfizer Incorporated, New York, NY, USA) 0.125–64 μg/mL; ITC (Pfizer Incorporated, New York, NY, USA), and AMB (Sigma Chemical Corporation, St. Louis, MO, USA) 0.015–8 μg/mL. The inocula of all strains tested were obtained from 48 h cultivation in Sabouraud broth at 30°C and an initial cellular suspension in saline solution equivalent to the 0.5 MacFarland standard, determined spectrophotometrically at 530 nm. Then, two serial dilutions were made, the first in saline solution (1:100) and the second in RPMI (1:20), in order to obtain a final concentration of 10^3^ cells/mL. Susceptibility to antifungal agents was evaluated by broth microdilution, as recommended within the document CLSI M27-A3 (CLSI, 2008). Aliquots of 100 μL of the final inoculum solution were dispensed in microtiter plates of 96 wells containing 100 μL of various concentrations of the tested drugs. Finally, the plates were incubated at 37°C and test reading taken after 72 h incubation. All strains were tested in duplicate. MIC was defined for azoles to the lowest drug concentration which showed approximately 50% reduction in turbidity as compared to the positive control well. For AMB, the MIC was defined as the lowest concentration able to inhibit any growth visually perceptible (CLSI, 2008; CLSI, 2012). Epidemiological cutoff values (ECVs) were used for *C. deuterogattii* (clade I) and *C. neoformans* (clade II). The ECV determines the upper limit of wild-type distribution (without intrinsic or acquired resistance mechanisms), distinguishing from non-wild-type isolates harboring intrinsic or acquired resistance mechanisms (CLSI, 2020).

### Statistical Analysis

Data were analyzed using the statistical software “Graph Pad version 8.0”. Results were presented as mean ± standard deviation, and differences were analyzed by the Mann–Whitney test. For all the analyses, P was considered a default value of 0.05 and the confidence interval of 95%. In addition, the values obtained for some of the virulence attribute tests *in vitro* were divided onto tertile categories as weak, moderate or strong producers.

## Results

### Phenotypic Preliminary Identification of the Strains Used in the Present Study

All the isolates produced encapsulated blastoconidia, did not ferment glucose and were able to hydrolyze urea. Some isolates, but not all of them, produced melanin, when grown on NSA. Therefore, they were presumptively identified as *Cryptococcus* spp. and related genera.

### Molecular Identification of the Strains Used in the Present Study

For molecular species identification, DNA fragments of the ITS region were amplified and DNA sequences obtained were sent to the GenBank genome database at the NCBI website (http://ncbi.nlm.nih.gov) using BLAST to compare gene sequences. All the *Cryptococcus gattii*/*neoformans* strains were unambiguously identified by sequencing, as follows: 45 as *C. neoformans* (formerly C. *neoformans* var. *grubii*) and 20 as *C. deuterogattii* (formerly *C. gattii* VGII genotype). It is important to recognize that the identification of these cryptococcal strains was further confirmed with the Multi-Locus Sequence Typing (MLST) consensus scheme of seven genetic loci (unpublished data). The same trend of accurate identification was also observed for *P. laurentii* (10 isolates). Nevertheless, for the other non-*Cryptococcus gattii*/*neoformans* species complex strains, a less precise identification was observed. For some of the strains (n=20), BLAST comparisons revealed 100% identity (E-value of 0) with three different *Naganishia* spp. (*N. liquefasciens*/*N. albidosimilis*/*N. diffluens*), while 10 of them were 99.8% identical to N. *albida* and *N. adeliensis*).

### Phylogenetic Analysis

**Figure 1:**
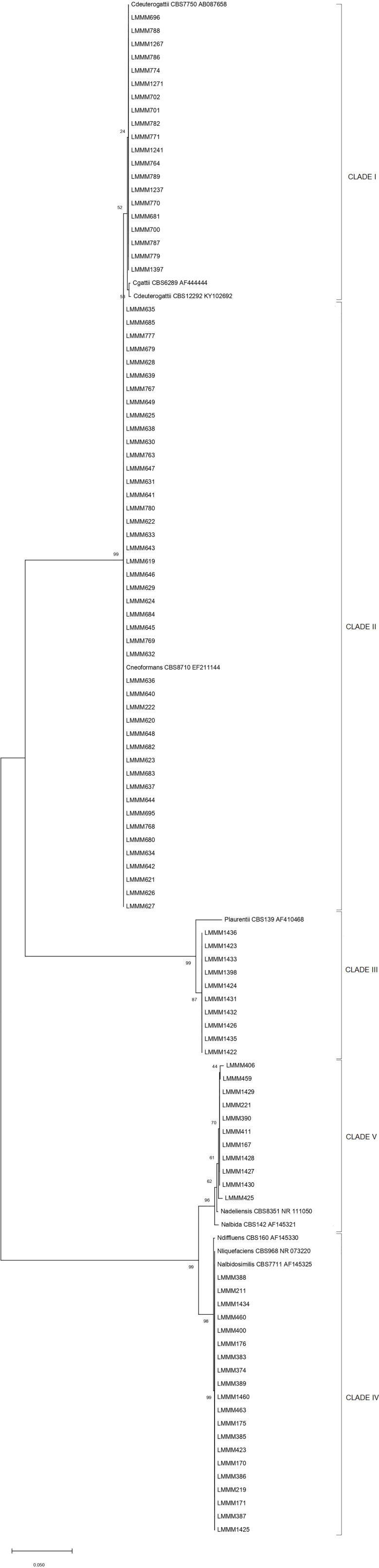
Neighbor-Joining phylogenetic tree of rDNA ITS sequences from 106 clinical and environmental isolates and 10 type strain sequences from *Cryptococcus* spp. and associated genera. Bootstrap values are depicted in all nodes (bt). The scale bar represents the number of base substitutions per site.

Phylogenetic analysis confirmed the species identification of 20 isolates as *C. deuterogattii* with bt of 54 (Clade I). The other isolates were confirmed as *C. neoformans* (45) and *P. laurentii* (10), respectively (Clades II and III), with high bt values (99). All the other yeast isolates were identified as *Naganishia* spp. due to low confidence values in the analysis to confirm BLASTn species identification. Twenty isolates belong to the *N. liquefasciens/N. albidosimilis* clade (bt=99), excluding *N. diffluens* from the most probable species (clade IV), while 11 isolates were identified within *the N. adeliensis/N. albida* clade, grouping closer to the first species (bt=62; clade V; **Figure 1**).

### Colony Phenotypes and Melanin Production of the Strains Used in the Present Study

**Figure 2:**
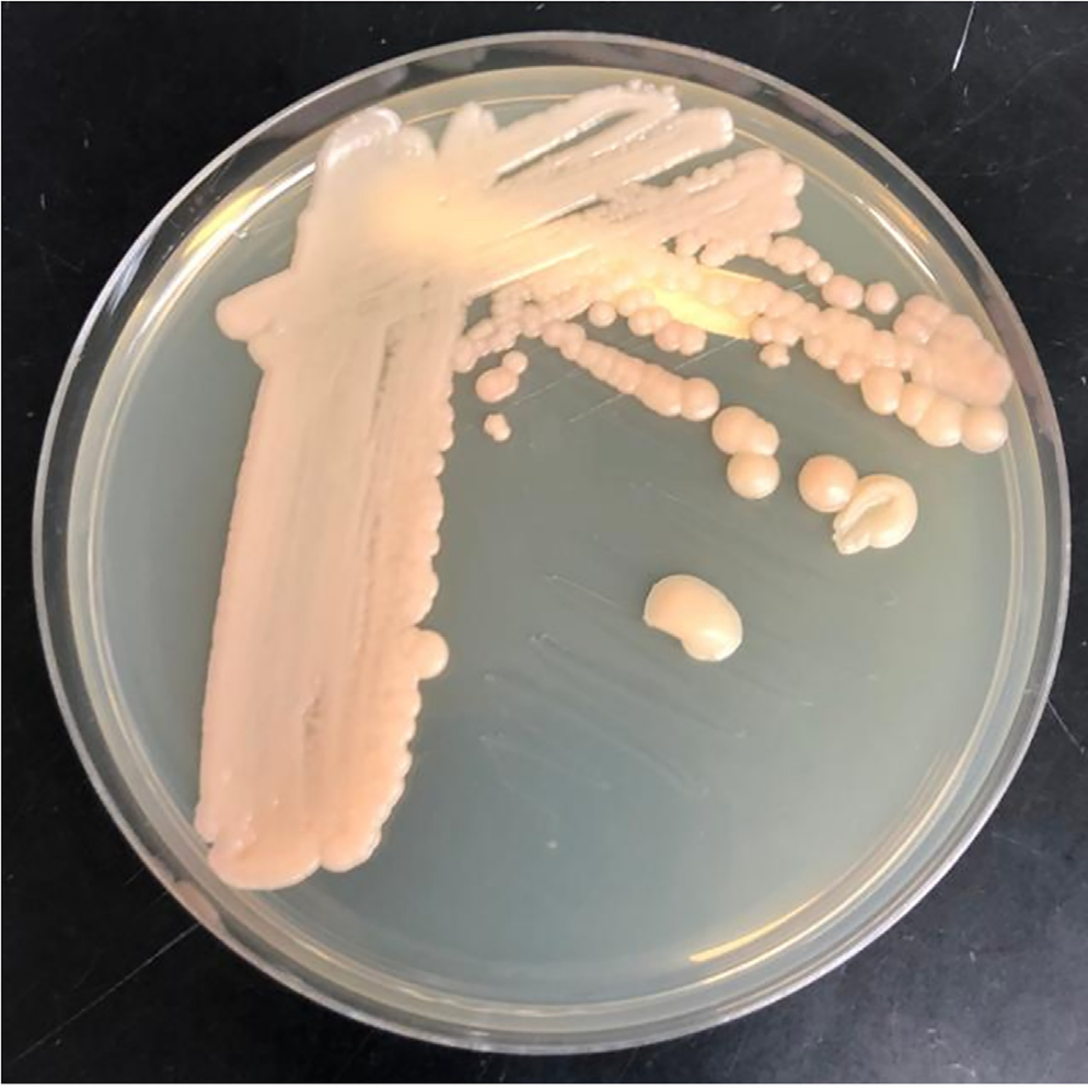
Phenotypic characteristics of *Naganishia* sp. after 48 h of incubation at 30° in YPD medium showing creamy colonies with an unusual pink pigment.

*Cryptococcus gattii*/*neoformans* species complex (clades I and II and *N. adeliensis*/*N*. *albida* (clade V) Chromagar *Candida* colony colors ranged from pale pink to purple (mainly pink for *C*. *neoformans* isolates), while all the isolates belonging to *P*. *laurentii* (clade II) and *N*. *liquefasciens/N. albidosimilis* (clade IV) were dark purple. All non-*Cryptococcus gattii*/*neoformans* species complex isolates (clades III to V) grew very poorly on Chromagar *Candida* (incubation at 37°C), whereas the opposite growth happened to clade I and II isolates (*Cryptococcus gattii*/*neoformans* species complex) All of the strains showed creamy to mucoid colony growth phenotypes when grown on YPD at 30°C, with all C. *neoformans* isolates being highly mucoid. Of note, strains belonging to clades III to V (non- *Cryptococcus gattii*/*neoformans* species complex) produced occasionally a pink pigment when grown on this culture medium (**Figure 2**). Approximately,67% of the isolates belonging to clades I and II produced dark colonies (scores 3 to 4) on NSA, while the other isolates (clades III to V) produced either light brown colonies (80.5%; score 1) or did not produce melanin at all (14.6%; score 0), except for isolates LMMM1398 (clade III), LMMM1460 (clade IV) and LMMM221 (clade V) colonies which were classified as score 3. Interestingly, these strains were non-*Cryptococcus gattii*/*neoformans* species complex strains obtained from patients (CSF samples) and not the environment (**Table 1**).

### *Cryptococcus* spp. and Related Genera Phospholipase Production

**Figure 3:**
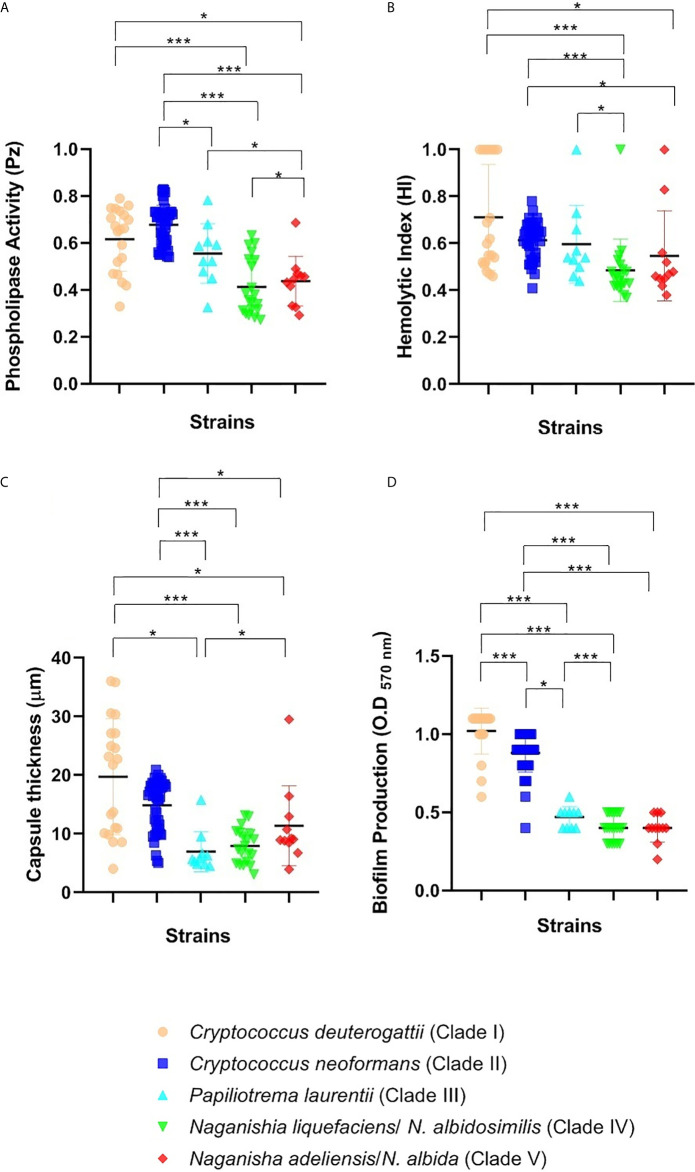
Virulence factors attributes of clinical and environmental strains of *Cryptococcus* spp. and related genera. **(A)** Phosphoplipase zone determined after incubating cells on egg yolk agar medium at 30°C, for 72 h. **(B)** Hemolytic index after incubation on SDA supplemented with sheep’s blood and glucose at 37°C for 48 h, 5% CO_2_. **(C)** Capsule thickness determined after cells incubation in 10% fetal bovine serum in PBS, incubated for 24 h at 37°C in an atmosphere of 5% CO_2_ and **(D)** Biofilm formation induced after cells incubation in 96-wells microtiter plates containing BHI medium at 37°C, for 48 h. Each bar represents mean ± standard deviation of the triplicate obtained for each isolate. *P < 0.05; ***P < 0.001.

Phospholipase production was detected in all of the isolates (100%) evaluated. However, the levels of enzyme production varied among strains of each species. Phospholipase activity is inversely proportional to Pz, where values equal to 1 mean negative activity. The enzyme production ranged from 0.27 ± 0.02 (LMMM460; *P*. *laurentii;* clade III) to 0.83 ± 0.04 (LMMM644; *C. neoformans* clade II). Most of *Cryptococcus gattii*/*neoformans* species complex (clades I and II strains) were considered weak (60%) to moderate phospholipase producers (35.4%; **Table 1** and **Figure 3**), while 51.2% of non- *Cryptococcus gattii*/*neoformans* species complex (clades III to V strains) were considered strong phospholipase producers and 41.5% showed moderate production of this enzyme. These data were reinforced with average value comparisons for the production of this enzyme, which showed significantly different results between *C. gattii*/*neoformans* species complex strains (clades I and II) and the other strains belonging to non- *Cryptococcus gattii*/*neoformans* species complex (clades III to V; **Figure 3**). Of note, the average value of *P. laurentii* (clade III) strains for phospholipase production was also considered lower than the results found for *N*. *liquefasciens/N. albidosimilis* (clade IV) and *N. adeliensis*/*N*. *albida* (clade V)strains (P<0.05; **Figure 3**). Our three clinical non-*C. gattii*/*neoformans* species complex isolates were considered strong (*P. laurentii*; clade III; LMMM1398; 0.45 ± 0.05 and *N. adeliensis*/*N*. *albida*;clade V; LMMM1429 0.45 ± 0.015) to moderate producers (*N*. *liquefasciens/N. albidosimilis*; clade IV; LMMM1460; 0.60 ± 0.09; **Table 1** and **Figure 3**).

### Production of Hemolysins by *Cryptococcus* spp. and Related Genera

Almost 91% of the isolates were able to produce hemolysis. HI is also inversely proportional to the diameter of the halo, where values equal to 1 mean negative activity. Most of the isolates in the present study were able to show medium (41.5%) to large halos (38.7%), meaning low to moderate HI. Interestingly, 7 C*. deuterogattii* (clade II) isolates did not produce halos suggesting that the enzyme(s) was not produced. The average values for non-*C. gattii*/*neoformans* species complex strains (clades III to V) of hemolysin production was significantly higher than those of *Cryptococcus gattii*/*neoformans* species complex (clades I and II) isolates (P<0.05; **Figure 3**). Most clade I and II strains were considered moderate hemolysin producers (53.8%) while larger halos (strong hemolysin production) were observed for 65.9% of clades III to V strains, being considered strong hemolysin producers, including all three clinical isolates within these clades. The average value for *P*. *laurentii* (clade III) strains hemolysin production was lower than the results found for *N*. *liquefasciens/N. albidosimilis* (clade IV) and *N. adeliensis*/*N*. *albida* (clade V)isolates (P<0.05; **Figure 3**). In addition, there was no statistically significant difference between *P*. *laurentii* (clade III) expression of this virulence factor *in vitro* and *C. gattii*/*neoformans* species complex (clades I and II)isolates. The isolates with the highest hemolytic activity was LMMM383, belonging to *N*. *liquefasciens/N. albidosimilis* (clade IV) (0.37 ± 0.06; **Table 1**).

### Determination of Capsule Thickness in *Cryptococcus* spp. and Related Genera

There was a huge interspecies variation in average values of capsule thickness among certain isolates of different clades. It ranged from 4.1 ± 1.27 µm (LMMM1397) to 36.07 ± 9.33 µm (LMMM787). Interestingly, both of these isolates belonged to *C. deuterogatti* (clade I) showing wide strain variation. When this species was analyzed separately, it was observed that this species contained the isolates with greatest capsule thickness (LMMM1241; 35.87 ± 7.33 µm and LMMM782; 30.61 ± 4.55 µm), and several others with sizes above 20 µm were observed in this species. Furthermore, there was not a significant difference when the average values for the two clades (I and II) were compared, due to the enormous variation in measurements found within *C. deuterogattii* (clade I) strains, despite the observation that the greatest capsule thickness observed for C. *neoformans* (clade II) strains was 20.95 ± 5.28 µm (LMMM620; **Table 1**). All the other isolates belonging to the non- *Cryptococcus gattii*/*neoformans* species complex (clades III to V) had significantly smaller capsules when compared to *Cryptococcus gattii*/*neoformans* species complex strains (clades I and II) and no differences in average sizes were found among them (**Figure 3**).

### Evaluation of Biofilm Formation in *Cryptococcus* spp. and Related Genera

All the isolates were able to form biofilms on polystyrene microtiter plates. However, there was a large variation in biofilm biomass among the isolates. The OD570nm readings ranged from 0.21 ± 0.03 (*N. adeliensis*/*N*. *albida*; clade V; LMMM406) to OD570nm of 1.14 ± 0.06, a *C. deuterogattii* (clade I) isolates (clade I; LMMM700 (P<0.05; **Table 1** and **Figure 3**). In fact, this was the unique virulence factor expressed *in vitro* that showed a significant difference between *C. deuterogatti* (clade I) and *C*. *neoformans* (clade II) isolates. For instance, 90% of *C. deuterogattii* (clade I) isolates were considered strong biofilm producers, while 75.6% of *C. neoformans* (clade II) isolates were included in this category. Taken together, 80% of *Cryptococcus gattii*/*neoformans* species complex isolates (clades I and II) were considered strong biofilm producers. The average values for clades I and II OD570nm readings were greater than the results found for the within the non- *Cryptococcus gattii*/*neoformans* species complex (clades III to V; P<0.05; **Figure 3**). Furthermore, the vast majority of isolates belonging to clades I and II were not considered weak biofilm producers (except for LMMM644, a *C*. *neoformans* (clade II) strain; OD570nm of 0.45 ± 0.09). In contrast, the non-*C. gattii*/*neoformans* species complex strains (clades III to V) had no isolates identified as high biofilm producers. All *N*. *liquefasciens/N. albidosimilis* (clade IV) and 95% of *N. adeliensis*/*N*. *albida* (clade V strains were actually considered weak biofilm producers. In addition, 60% of *P*. *laurentii* strains (clade III) showed low biofilm formation, and the other 40% were considered moderate biofilm producers (**Table 1**). If we compare the average values of OD570nm readings among all three clades, *P*. *laurentii* (clade III) showed a trend for higher biofilm production compared to the other 2 clades (IV and V) and this difference was statistically significant between clades III and IV (**Figure 3**). Interestingly, the clinical isolates belonging to the non- *Cryptococcus gattii*/*neoformans* species complex (clades III to V) were also considered weak to moderate biofilm producers (**Table 1** and **Figure 3**).

### Antifungal Susceptibility Testing for *Cryptococcus* spp. and Related Genera

All MIC values obtained by the reference strains were compatible with the values expected by the CLSI methodology, assuring the reliability of the results obtained for the isolates tested. MIC ranges and geometric means (GMs) for all the antifungal drugs tested are displayed in **Table 2**. Because there are no breakpoints established to *Cryptococcus* spp. and related genera, we have included ECVs, according to the CLSI M59-Ed3 document for *C. deuterogattii* (clade I) and *C. neoformans* (clade II) strains (species that ECVs are available).

**Table 2:** Geometric means, MIC range, MIC50 and MIC90 of clinical and environmental isolates of *Cryptococcus* spp. and related genera obtained from patients and pigeon droppings in Northeast Brazil.

Species	Drug	GM*	MIC** range	MIC_50_	MIC_90_
*C. deuterogattii* (clade I; n = 20)	Fluconazole	5.28	0.5 - 16	8	8
	Itraconazole	0.05	0.03125 - 0.25	0.03125	0.125
	Amphotericin B	0.76	0.25 - 2	1	1
*C. neoformans* (clade II; n = 45)	Fluconazole	2.89	0.25 - 16	4	8
	Itraconazole	0.04	0.03125 - 0.125	0.03125	0.0625
	Amphotericin B	0.59	0.25 - 2	0.5	2
*P. laurentii* (clade III; n = 10)	Fluconazole	1.74	0.0 - 8	2	4
	Itraconazole	0.10	0.03125 - 0.25	0.125	0.25
	Amphotericin B	1.32	1 - 2	1	2
*N. liquefasciens*/*N. albidosimilis* (clade IV; n = 20)	Fluconazole	2.22	0.25 - 16	2	16
	Itraconazole	0.06	0.03125 - 0.25	0.0625	0.125
	Amphotericin B	0.84	0.125 - 2	1	2
*N. albida*/*N. adeliensis* (clade V; n = 11)	Fluconazole	3.11	0.5 - 16	4	8
	Itraconazole	0.13	0.03125 - 0.25	0.125	0.25
	Amphotericin B	0.94	0.5 - 2	1	1

^*Geometric Mean,**Minimal Inhibitory Concentration.^

Most strains had low MICs to fluconazole, while a few of them showed increased MICs. Two out of 45 (4.4%) *C. neoformans* strains (LMMM629 and LMMM631) where considered non-wild-type. All *P. laurentii* isolates had low MICs to fluconazole. Four out of 20 (20%) clade IV strains and a single *N*. *albida* isolates (LMMM1430; clade V; 9.1%), had MICs ≥16 µg/mL (**Table 2** and **Supplementary Table 1**).

All the strains tested had MICs below the ECVs established for *C. deuterogattii* (clade I) and *C. neoformans* (clade II) to itraconazole. Some strains belonging to the non-*Cryptococcus gattii/neoformans* species complex (clades III to V) had higher MICs, when compared to clade I and II strains MICs ≥ 0.25 µg/mL (**Table 2** and **Supplementary Table 1**).

Several isolates in the present study were considered of non-wild-type phenotype to amphotericin B. For instance, in *C. deuterogattii*, 2 out of 20 strains (LMMM1271 and LMMM 774; 10%) had MICs above the ECV; in *C*. *neoformans*, this phenomenon happened with 18 out of 45 (40%) strains. All *P. laurentii* (clade III) and most of *N. liquefasciens*/*N. albidosimilis* (clade IV) and *N. adeliensis*/*N. albida* (clade V) strains showed MICs equal to or above the ECV established for *C. deuterogattii* (clade I; ECV=1 µg/mL) and *C. neoformans* (clade II; ECV=2 µg/mL; **Table 2** and **Supplementary Table 1**).

## Discussion

Our molecular identification and phylogenetic analysis revealed five different *Cryptococcus* spp. and related genera clades. Regarding the *C. gattii*/*neoformans* species complex isolates (clades I and II), which comprised the vast majority of the clinical isolates, BLAST searches showed that our *C. gattii* isolates (clade I) belonged to the VGII genotype, named as *C. deuterogatti* (Hagen et al., 2015). This is the main species recovered in Brazil from both environmental and clinical samples (Dos Freire et al., 2012; Maruyama et al., 2019; Santos Bentes et al., 2019). Following the same trend of other studies performed in Brazil (Cogliati, 2013; Alves et al., 2016; Ferreira-Paim et al., 2017), our *C. neoformans* strains were all considered the former *C*. *neoformans* var. *grubii*, but because of the recent recognition as a separate species in the *C.gattii*/*neoformans* species complex (Hagen et al., 2015), we will use the nomenclature *C*. *neoformans*, as recommended by Hagen et al., (Hagen et al., 2017).

Regarding the non-*C. gattii*/C. *neoformans* species complex (clades III to V) isolates identification, we faced a limitation using ITS region sequencing, particularly among *Naganishia* spp. (clades IV and V) isolates. When our sequences were submitted to GenBank, the final identification was not precise, because they were considered identical to three different species as follows: N. *liquefaciens*, *N.albidosimilis* and *N*. *diffluens* (clade IV). However, our phylogenetic analysis excluded *N*. *diffluens*. The same level of indistinguishability was found for *N*. *albida*/*adeliensis* (clade V). Furthermore, all of them were previously considered *Cryptococcus* spp., but Liu et al. (Liu et al., 2015) reclassified several Tremellomycetes isolates, based on the results of phylogenetic analyses from a seven-genes dataset, including the large subunit ribosomal DNA (LSU rDNA locus). Because we have only used a single locus in our study (ITS region), we labeled our isolates as *Naganishia* spp. only and have submitted the sequences to the GenBank database as such. Further typing using DNA sequence of different loci will be needed for accurate species identification.

Similar to our findings, Brito et al., (Brito et al., 2019) reported the recovery of *P*. *laurentii*, *N*. *albida* and *N*. *liquefaciens* among other former *Cryptococcus* spp. from bird excreta, collected outside a Brazilian University hospital, but no *C*. *neoformans* strains were found. *P*. *laurentii* was also recovered from bird droppings samples in Brazil, together with *C*. *neoformans* in the same samples (Andrade-Silva et al., 2010; Ferreira-Paim et al., 2011). *N*. *albida*, *N. uzbekistanensis*, *N. adeliensis* and *C*. *neoformans* were isolated from pigeon nests. *N. adeliensis* was the only species isolated from Eucalyptus trees in Ilam, Iran (Kamari et al., 2017).

Although most human infections and specifically, those related to the CNS are caused by *C. gattii*/*neoformans* species complex isolates, less common former *Cryptococcus* spp. have been reported to cause invasive disease due to *P. laurentii* and *N. albida* (Cheng et al., 2001; Averbuch et al., 2002; McCurdy and Morrow, 2003; Shankar et al., 2006; Khawcharoenporn et al., 2007; Furman-Kuklinska et al., 2009; Gullo et al., 2013; Neves et al., 2015). A systematic review performed by Londero et al., (Londero et al., 2019) reported 35 cases of deep-seated infections due to *P*. *laurentii*. In this study, 16.1% of cases were catheter-related, 67.7% had positive blood (54.8%) or CSF (12.9%) cultures. Most patients were immunosuppressed and 48.4% of these patients had neoplasia as an underlying disease. Choe et al. (Choe et al., 2020) reported a case series of 20 patients with *N*. *albida* (or the former *C*. *albidus* infections), and these patients had high mortality rates (40%) despite antifungal treatment. The isolates came from the bloodstream in 9 cases, CNS in 6 cases, lower respiratory tract in 5 cases, and a single sample from peritoneal fluid. Seven cases of non-invasive infections were also reported, including colonization of the skin and fungal keratitis. Furthermore, a case of catheter-related fungemia due to *N*. *liquefaciens* has been described in a 71-year-old man with B-cell lymphoma (Takemura et al., 2015). In addition, a CSF mixed infection caused by *N. liquequefaciens* and *Mycobacterium tuberculosis* species complex has been reported in an HIV-positive female patient who died despite antifungal and antibacterial therapy. The final identification of this isolate was only possible with the sequencing of the D1/D2 domains of the large subunit of the rRNA region, since ITS rDNA sequencing was not able to differentiate between *N*. *liquefaciens* and *N. albidosimilis* (Conde-Pereira et al., 2015) and similar to our findings.

All *C. gattii*/*C*. *neoformans* species complex strains (clades I and II) isolates were able to grow at 37°C, but the high temperature growth phenotype was not observed for non-*C. gattii*/*C*. *neoformans* species complex strains (clades III to V), even if they were clinical isolates (LMMM1398, 1460 and 221). It is widely documented that the ability to grow at human physiological temperature is an important virulence factor for *Cryptococcus* spp. (Zaragoza, 2019). Nevertheless, other opportunistic fungi such as *Rhodotorula* spp. grow poorly *in vitro* in temperatures above 33°C, and yet, can cause human infections such as fungemia (Ioannou et al., 2019) and may also belong to the human gut microbiota (Borges et al., 2018).

It is very clear that *C. gattii*/*neoformans* species complex isolates (clades I and II), in general, show more similar virulence phenotypes among each other when compared to non-*C. gattii*/*C*. *neoformans* species complex isolates (clades III to V) and vice versa. However, occasionally, *P*. *laurentii* (clade III) strains do behave closer to *C. gattii*/*neoformans* species complex isolates (clades I and II) such as with phospholipase and hemolysins production. While clades I and II strains have thicker capsules, produced more melanin and formed greater biofilms, clades III to V strains were able to produce the enzymatic activities of phospholipase and hemolysins. However, it is important to emphasize that because we do not have a balanced number of clinical and environmental strains for each clade, comparisons were performed independently of the source of the strain isolation We recognize that if more clinical strains of *P. laurentii* and *Naganishia* spp. (clades III to V) or environmental strains of *C. gattii*/*neoformans* species complex isolates (clades I and II) were included, our results could have been different because of strain adaptations to different ecological niches. Therefore, we recognize this fact as a limitation of our study.

Ferreira-Paim et al. (2012) were the first to describe hemolysin production by the former *C*. *laurentii* isolates obtained from pigeon droppings. The fact that non-*C. gattii*/*neoformans* species complex strains are able to efficiently produce hemolysins may partially explain the reason why these species are more frequently described in cases of fungemia, rather than meningeal or deep-seated tissue infections (Londero et al., 2019). Conversely, phospholipase production has been largely reported for *C. gattii*/C. *neoformans* species complex strains (Pini et al., 2017). This enzyme in C. *neoformans* is thought to trigger capsule enlargement, reduce phagocytosis by macrophages (Chrisman et al., 2011) and secreted phospholipase B (PLB) activity favors the survival and replication of *C*. *neoformans* in macrophages *in vitro*, but it seems that this enzyme is not directly involved in the establishment of CNS infections (Santangelo et al., 2004). Interestingly, Pedroso et al. (2009) reported greater phospholipase activity in *C. albidus* than in *C. laurenttii* isolates recovered from several organs in a murine model of infection, consistent with our findings. However, it appears that these extracellular enzymes may be critical in breaking down the environmental milieu rather than participating in mammalian host tissue invasion.

Most of the clinical isolates when grown on NSA had colonies that were darkly pigmented (scores 3 to 4), including the non-*C. gattii*/C. *neoformans* species complex clinical isolates (LMMM1398, LMMM1460 and LMM221) and supports the importance of this as a mammalian virulence factor. Furthermore, melanin production is crucial for *Cryptococcus* spp. virulence, since mutants unable to produce this pigment have a significant attenuation in virulence (Salas et al., 1996). Melanin confers resistance to oxidative stress, heat and ionizing radiation (Zaragoza, 2019), besides impairing proper antifungal drug actions (Nosanchuk and Casadevall, 2006).

In our biofilm formation experiments, *C. deuterogatti* (clade I) was more efficiently able to form biofilm than C. *neoformans* (clade II) strains. Tavares et al. (2019) recently investigated *C. gattii* (VGI) kinetics of biofilm formation and described its structure as a dense network of yeast cells deeply encased in an extracellular polymeric matrix (Tavares et al., 2019). In terms of clinical significance, *C. gattii* species are frequently described in the immunocompetent host, cause more damage to the lungs and frequently produce cryptococcomas, which are considered biofilm-like structures (Chen et al., 2014; Aslanyan et al., 2017; Rodriguez-Goncer et al., 2018). In our study, there was also trend of greater biofilm formation in *P*. *laurentii* (clade III*)* than in *Naganishia* spp. (clades IV and V), but the three clades together were considered significantly less effective in biofilm formation than *C. gattii*/*C*. *neoformans* species complex strains (clades I and II). However, *P*. *laurentii* biofilm was recently described to have dense growth and colonization with extensive polymeric substances around the yeast cells (Ajesh and Sreejith, 2012).

*C. deuterogatti* (clade I) strains showed a large variation in capsule sizes, with some of the isolates possessing the largest capsules in the present study. Giant yeast cells (capsule thickness ≥ 15 µm) and variation in capsule thickness are more common in *C. gattii* complex than in the C. *neoformans* complex. However, both “large” and “small” capsular phenotypes are important in different stages of infection (early and late, respectively), reinforcing the advantage of strains with plasticity in capsule and cell sizes to cause cryptococcosis (Fernandes et al., 2018). The non-*Cryptococcus gattii*/*neoformans* species complex strains (clades III to V) had markedly thinner capsules. However, Araujo et al., (Araujo et al., 2017) described that polysaccharides of *C*. *liquefaciens* have strikingly similar ultrastructural and biological properties to those of C. *neoformans*, and found that this species led to mortality rates similar to *C. neoformans* in a virulence model with the larvae of *Galleria melonella*, reinforcing its pathogenic potential.

There are no antifungal breakpoints yet established for Cryptococcal species so discussions about drug susceptible and resistant isolates need to wait until breakpoints are determined. However, it is clear from our results most strains fit under the ECVs established for *C. deuterogattii* (clade I) and *C. neoformans* (clade II) and surely do not possess high-level resistance since azole MICs are within achievable drug levels in the blood. On the other hand, the isolates have some variability in their azole susceptibility. For the polyene *in vitro* activity, some of the MICs are relatively high and this may be more *in vitro* media importance rather than clinical importance. In a Chinese multicenter study almost 24% of 312 *C. neoformans* strains had non-wild-type phenotypes to fluconazole with 5 isolates possessing had MICs≥ 32 µg/mL (Ajesh and Sreejith, 2012) but Pfaller et al. with over 1,811 clinical isolates reported only 1% with very high MICs. Ferreira and Pahim (Ferreira-Paim et al., 2012) found that 71% of 38 environmental *C. laurentii* isolates had elevated azole MICs and Kantarcioglu et al. (2009) showed decreased susceptibility of 7/11 C. *diffluens* to both azoles and polyenes. Both our data and others suggest that azoles and polyenes will likely have clinical activity against both species complexes, the *C. gattii*/*neoformans* and non-*C. gattii*/*neoformans* species complex. However, some isolates push toward the higher antifungal MICs especially with the non-*C. gattii*/*neoformans* species complex strains which also possess less clinical treatment experience. Therefore, assessment of *in vitro* susceptibility for these isolates may be helpful to the clinician during clinical management.

## Conclusions

In conclusion, we have shown different phenotypic traits between *C. gattii*/*neoformans* species complex (clades I and II) and other related genera such as *Papiliotrema* (clade III) and *Naganishia* (clades IV and V). In addition, *P*. *laurentii* (clade III) shows phenotypic properties more similar to *C. gattii*/C. *neoformans* species complex isolates (clades I and II), when compared to *Naganishia* spp (clades IV and V). But *Naganishia* spp. produced higher enzymatic levels of phospholipase and hemolysins. Furthermore, *C. gattii*/C. *neoformans* species complex (clades I and II) isolates had markedly larger capsules, produced more biofilm biomass and melanin. In addition, more isolates belonging to non- *Cryptococcus gattii*/*neoformans* species complex (clades III to V) possessed higher MICs to fluconazole, itraconazole and amphotericin B than those belonging to the *C. gattii*/*neoformans* species complex (clades I and II). However, 40% of *C. neoformans* strains had MICs equal to or above the ECV to amphotericin B. *C. deuterogattii* and C. *neoformans* showed very similar phenotypes among themselves. However, *C. deuterogatti* strains (clade I) produced statistically significant more biofilm biomass than C. *neoformans* (clade II). Another interesting observation is that all the non-*C. gattii*/*neoformans* clinical isolates (clades III to V) produced darker colonies, suggesting higher melanin production, when compared to their environmental counterparts. More isolates are needed to determine if there is an association of melanin production with non-*C. gattii*/*C*. *neoformans* clinical isolates. These findings, together with the fact that *P*. *laurentii* and *Naganishia* spp. strains were collected outside of a University Hospital, could be important for environmental to patient transmission that produces nosocomial infections especially in immunocompromised patients.

## Data Availability Statement

The original contributions presented in the study are included in the article/**Supplementary Material**, further inquiries can be directed to the corresponding author/s.

## Author Contributions

Conceptualization, GC. Methodology, GC, LO, LP, MM, DT, and JT. Software, AP and WS. Formal analysis, WS and GC. Investigation, EP, RN, RL, and TB. Resources, WS and AP. Data curation, LO, WS, and AP. Writing—original draft preparation, GC, JP, and JT. Writing review and editing, GC, JP, and JT. Supervision, EM, RN, RL and TB. Project administration, GC. Funding acquisition, GC and JP. All authors contributed to the article and approved the submitted version.

## Funding

This research was funded by Public Service Grants (USA) from NIAID (AI-73896 and AI-93257) and from the “Coordenação de Aperfeiçoamento de Pessoal de Nível Superior (CAPES)”– Finance Code 001, Brazil (Ministry of Education). L. S. S. O. received a scholarship from CAPES. GC, RL, and RN are research productivity fellows of the “Conselho Nacional de Desenvolvimento Científico e Tecnológico (CNPq)”, Brazilian Ministry of Science and Technology.

## Conflict of Interest

The authors declare that the research was conducted in the absence of any commercial or financial relationships that could be construed as a potential conflict of interest.
