# Supplementary material for: Comparison of Cryptococcus gattii/neoformans Species Complex to Related Genera (Papiliotrema and Naganishia) Reveal Variances in Virulence Associated Factors and Antifungal Susceptibility
Source: Front Cell Infect Microbiol. 2021 Jul 1;11:642658. doi: 10.3389/fcimb.2021.642658 (PMC8281300; doi:10.3389/fcimb.2021.642658)
Supplement: Supplementary file 1 [file Table_1.docx]

Supplementary Table S1. Antifungal susceptibility testing of clinical and environmental isolates of *Cryptococcus* spp. and related genera obtained from patients and pigeon droppings in Northeast Brazil.

| **Minimal Inhibitory Concentrations (μg/mL) for the antifungal drugs tested** | | | |
| --- | --- | --- | --- |
|  | Fluconazole | Itraconazole | Amphotericin B |
| Strain name |  |  |  |
| *C. deuterogattii* (clade I) |  |  |  |
| LMMM696 | 4 | 0.03125 | 0.5 |
| LMMM700 | 8 | 0.25 | 0.5 |
| LMMM701 | 8 | 0.125 | 0.5 |
| LMMM702 | 0.5 | 0.125 | 0.5 |
| LMMM764 | 16 | 0.03125 | 1 |
| LMMM1271 | 1 | 0.125 | 2* |
| LMMM681 | 8 | 0.125 | 1 |
| LMMM1241 | 8 | 0.125 | 1 |
| LMMM1267 | 8 | 0.03125 | 1 |
| LMMM1397 | 8 | 0.125 | 1 |
| LMMM1237 | 8 | 0.03125 | 1 |
| LMMM770 | 4 | 0.03125 | 0.5 |
| LMMM771 | 4 | 0.03125 | 1 |
| LMMM774 | 2 | 0.03125 | 2* |
| LMMM779 | 8 | 0.03125 | 0.5 |
| LMMM782 | 8 | 0.03125 | 1 |
| LMMM786 | 8 | 0.03125 | 0.5 |
| LMMM787 | 8 | 0.03125 | 1 |
| LMMM788 | 8 | 0.03125 | 0.5 |
| LMMM789 | 4 | 0.0625 | 0.25 |
| *C. neoformans* (clade II) |  |  |  |
| LMMM763 | 1 | 0.03125 | 1* |
| LMMM767 | 4 | 0.03125 | 2* |
| LMMM768 | 4 | 0.0625 | 0.25 |
| LMMM769 | 8 | 0.03125 | 2* |
| LMMM695 | 1 | 0.03125 | 1* |
| LMMM619 | 4 | 0.0625 | 2* |
| LMMM679 | 4 | 0.0625 | 2* |
| LMMM680 | 8 | 0.0625 | 0.25 |
| LMMM682 | 2 | 0.0625 | 1* |
| LMMM683 | 1 | 0.125 | 0.25 |
| LMMM684 | 0.5 | 0.125 | 2* |
| LMMM685 | 0.25 | 0.0625 | 1* |
| LMMM222 | 2 | 0.03125 | 0.5 |
| LMMM620 | 2 | 0.03125 | 0.25 |
| LMMM621 | 2 | 0.03125 | 0.5 |
| LMMM622 | 1 | 0.03125 | 1* |
| LMMM623 | 1 | 0.0625 | 0.5 |
| LMMM624 | 0.25 | 0.03125 | 0.25 |
| LMMM625 | 0.5 | 0.03125 | 0.25 |
| LMMM626 | 8 | 0.0625 | 0.25 |
| LMMM627 | 8 | 0.03125 | 0.25 |
| LMMM628 | 8 | 0.0625 | 0.25 |
| LMMM629 | 16* | 0.03125 | 0.5 |
| LMMM630 | 0.5 | 0.03125 | 0.5 |
| LMMM631 | 16* | 0.03125 | 0.5 |
| LMMM632 | 8 | 0.03125 | 0.5 |
| LMMM633 | 4 | 0.03125 | 0.5 |
| LMMM634 | 4 | 0.03125 | 0.5 |
| LMMM635 | 4 | 0.03125 | 0.5 |
| LMMM636 | 2 | 0.03125 | 0.5 |
| LMMM637 | 8 | 0.03125 | 1* |
| LMMM638 | 8 | 0.0625 | 1* |
| LMMM639 | 8 | 0.03125 | 1* |
| LMMM640 | 4 | 0.0625 | 0.25 |
| LMMM641 | 4 | 0.0625 | 0.5 |
| LMMM642 | 2 | 0.0625 | 0.5 |
| LMMM643 | 1 | 0.125 | 0.25 |
| LMMM644 | 4 | 0.03125 | 1* |
| LMMM645 | 4 | 0.03125 | 1* |
| LMMM646 | 4 | 0.03125 | 1* |
| LMMM647 | 8 | 0.03125 | 1* |
| LMMM648 | 8 | 0.03125 | 1* |
| LMMM649 | 4 | 0.125 | 0.5 |
| LMMM777 | 8 | 0.03125 | 0.25 |
| LMMM780 | 1 | 0.03125 | 0.5 |
| *P. laurenttii* (clade III) |  |  |  |
| LMMM1422 | 4 | 0.0625 | 1 |
| LMMM1423 | 4 | 0.125 | 2 |
| LMMM1424 | 1 | 0.125 | 1 |
| LMMM1426 | 0.5 | 0.25 | 1 |
| LMMM1431 | 4 | 0.25 | 2 |
| LMMM1432 | 2 | 0.0625 | 1 |
| LMMM1433 | 0.5 | 0.25 | 2 |
| LMMM1435 | 2 | 0.125 | 1 |
| LMMM1436 | 0.5 | 0.03125 | 2 |
| LMMM1398 | 8 | 0.03125 | 1 |
| *N. liquefasciens*/ *N. albidosimilis* (clade IV) |  |  |  |
| LMMM388 | 0.5 | 0.0625 | 1 |
| LMMM211 | 1 | 0.125 | 1 |
| LMMM1434 | 4 | 0.125 | 0.5 |
| LMMM460 | 16 | 0.25 | 2 |
| LMMM400 | 2 | 0.03125 | 0.125 |
| LMMM374 | 0.25 | 0.125 | 0.5 |
| LMMM383 | 1 | 0.03125 | 1 |
| LMMM385 | 1 | 0.03125 | 1 |
| LMMM386 | 2 | 0.125 | 2 |
| LMMM387 | 4 | 0.0625 | 2 |
| LMMM389 | 0.25 | 0.125 | 0.5 |
| LMMM423 | 16 | 0.03125 | 1 |
| LMMM170 | 2 | 0.03125 | 1 |
| LMMM171 | 8 | 0.03125 | 2 |
| LMMM175 | 2 | 0.125 | 1 |
| LMMM176 | 16 | 0.03125 | 1 |
| LMMM463 | 16 | 0.0625 | 0.125 |
| LMMM219 | 1 | 0.125 | 1 |
| LMMM1425 | 0.5 | 0.03125 | 2 |
| LMMM1460 | 4 | 0.03125 | 0.5 |
| *N. albida*/*N. adliensis*(clade V) |  |  |  |
| LMMM167 | 2 | 0.25 | 0.5 |
| LMMM1428 | 8 | 0.125 | 1 |
| LMMM1427 | 4 | 0.125 | 1 |
| LMMM1430 | 16 | 0.25 | 1 |
| LMMM425 | 2 | 0.125 | 1 |
| LMMM406 | 2 | 0.03125 | 1 |
| LMMM411 | 8 | 0.125 | 1 |
| LMMM390 | 1 | 0.125 | 1 |
| LMMM459 | 0.5 | 0.125 | 0.5 |
| LMMM1429 | 4 | 0.25 | 2 |
| LMMM221 | 4 | 0.0625 | 1 |

*Strains that were considered of non-wild-type phenotype according to the CLSI M59-3ed document.
